# The systematic approach to improving care for Frail Older Patients (SAFE) study: A protocol for co-designing a frail older person’s pathway

**DOI:** 10.12688/hrbopenres.12804.2

**Published:** 2018-05-08

**Authors:** Éidín Ní Shé, Mary McCarthy, Deirdre O'Donnell, Orla Collins, Graham Hughes, Nigel Salter, Lisa Cogan, Coailfhionn O'Donoghue, Emmet McGrath, John O'Donovan, Andrew Patton, Eilish McAuliffe, Diarmuid O'Shea, Marie Therese Cooney

**Affiliations:** 1 School of Nursing, Midwifery and Health Systems, University College Dublin, Dublin, D4, Ireland; 2 Older Person’s Empowerment Network and Patient and Public Involvement Representative in Healthcare at the Health Service Executive, Dublin, D8, Ireland; 3 St Vincent's University Hospital, Dublin, Dublin, D4, Ireland; 4 Royal Hospital Donnybrook, Dublin, D4, Ireland; 5 St Columcille's Hospital, Loughlinstown, County Dublin, Ireland; 6 Health Service Executive , Dun Laoghaire, County Dublin, Ireland; 7 School of Medicine, University College Dublin, Dublin, D4, Ireland

**Keywords:** Frailty, Co-design, Pathway, Public and Patient Involvement, Integrated Care, Geriatric, Delivery of Health Care, Older Adults

## Abstract

**Background**: Frailty is the age-accelerated decline across multiple organ systems which leads to vulnerability to poor resolution of homeostasis after a stressor event. This loss of reserve means that a minor illness can result in a disproportionate loss of functional ability. Improving acute care for frail older patients is now a national priority and an important aspect of the National Programme for Older People in Ireland. Evidence suggests that an interdisciplinary approach incorporating rapid comprehensive geriatric assessment and early intervention by an interdisciplinary team can reduces susceptibility to hospitalisation related functional decline. The aim of the Systematic Approach to Improving Care for Frail Older Patients (SAFE) is to develop and explore the process of implementing a model of excellence in the delivery of patient-centred integrated care within the context of frail older people’s acute admissions.

**Methods**: The SAFE study will employ a mixed methodology approach, including a rapid realist review of the current literature alongside a review of baseline data for older people attending the emergency department. Semi-structured interviews will be undertaken to document the current pathway. The intervention processes and outcomes will be jointly co-designed by a patient and public involvement (PPI) group together with the interdisciplinary healthcare professionals from hospital, community and rehabilitation settings. Successive rounds of Plan-Do-Study-Act cycles will then be undertaken to test and refine the pathway for full implementation.

**Discussion**: This research project will result in a plan for implementing an integrated, patient-centred pathway for acute care of the frail older people which has been tested in the Irish setting. During the process of development, each element of the new pathway will be tested in turn to ensure that patient centred outcomes are being realised. This will ensure the resulting model of care is ready for implementation in the context of the Irish health service.

## Introduction

There has been significant agreement within the literature that hospital admission is considered a health risk for frail older patients ^1^. Frail older people due to their loss of physiologic reserve across multiple organ systems, are vulnerable and can experience disproportionate loss of functional ability even when hospitalised for a relatively minor illness ^2^. Delays to the clinical decision-making process have been found to prolong hospital stays ^3, 4^ exacerbating the problem further. Providing a comprehensive multidisciplinary integrated care approach has been suggested as the best response to prevent functional loss among older patients ^5, 6^.

As older people have different healthcare requirements, the Irish healthcare system needs to adapt to meet these requirements, particularly as the demand will increase due to the aging population. For many older people in Ireland the emergency department (ED) is the ‘front door’ of entry to acute care. A recent Irish Department of Health special delivery unit report demonstrated that people aged over 65 year’s account for over one-third of ED attendances ^7, 8^. As this age group accounts for only 11.67% of the total population this indicates a disproportionate level of use of ED by the older population ^9^ and raises concern about whether services will be able to cope with rapidly increasing demand. Over the next 30 years, the over 80 population is set to rise dramatically, increasing from 128,000 in 2011 to 484,000 by 2046, under the positive migration assumptions ^10^. We can expect a continuing upward trajectory in ED attendances for the older person’s population ^11^

Although not an inevitable part of ageing, frailty is an increasingly common condition in older people. Frailty affects approximately 8–10% of people over the age of 65 and 25–50% of those aged 85 and over ^12^. Frailty considers the complex interplay of physical, psychological, social, and environmental factors and is associated with key clinical syndromes including loss of mobility, falls, confusion, incontinence and polypharmacy ^13– 15^. Frail older patients are particularly vulnerable to adverse effects of hospitalisation, including deconditioning, immobility, and the loss of independence ^16– 19^. A large systematic review demonstrated that frail elderly patients who received comprehensive geriatric assessment (GCA) had a significantly increased chance of being independent at 6 months’ follow-up ^3^. CGA is a multidisciplinary diagnostic process focused on determining a person’s medical, psychological, and functional capability, and assessing their social and environmental circumstances to develop a coordinated and integrated plan for treatment and long term follow up. Providing rapid access to consultant delivered CGA is one of the major elements of successful pathways for frail older people. These benefits were particularly significant when CGA was delivered to patients in specific geriatric units.

Recently, attention has focused on identifying the best pathways for treating frail older patients. Previous studies illustrate the importance of continuing organizational support, clinical champions who communicate regularly with decision makers, dedicated staffing, and ongoing data collection ^20, 21^. Acute frailty units are an exemplar of one approach to improve pathways of care of frail older people ^19^. They provide patients with rapid transfer to specialised geriatric units, consultant geriatrician delivered GCA, assessment by the full multidisciplinary team (IDT), twice daily IDT meetings, promotion of quick and safe discharge through expedited investigation, and rapid initiation of appropriate management plans. Improved links with the community provide for better support and follow-up post discharge ^22^. Several units in the United Kingdom have assessed the benefits of acute frailty units (AFUs) ^19, 23^. These have demonstrated reduced admissions, shorter length of stay, avoidance of admission and increased percentages of admission < 24hours to EDs ^3, 24– 26^. Some have also demonstrated reduced inpatient mortality and reductions in cost ^23^. This evidence suggests that AFUs represent an improved pathway for the care of frail older persons. However, very little information is available regarding implementation of strategies such as these in the Irish context ^11^. Significantly there is also very little evidence of involving or including public and patient involvement (PPI) in co-designing these types of interventions within the healthcare setting ^27, 28^. It is within this context that we have designed the Systematic Approach to Improving Care for Frail Older Patients (SAFE) to include PPI co-design of care pathways for acute frail patients.

## Overall study aim

We aim to collaborate with knowledge users in St Vincent’s University Hospital (SVUH), and with public and patient representatives, as well as community service providers, and rehabilitation facilities to develop and explore the process of implementing a model of excellence in the delivery of patient-centred integrated care within the context of acute presentations of frail older people. Specific objectives are to:

**1**. Assess current evidence of procedures in the acute care of frail older persons in SVUH and engage with key stakeholders, including patient on perceptions of current deficiencies.
**2**. Develop processes for patient centred integrated care for frail older patients admitted to acute hospitals which meet national policy document requirements and incorporate goals which are important from a patient perspective.
**3**. Explore the barriers and facilitators to effective implementation. Further refine the new model of care, through successive cycles of PDSA (plan, do, study act) assessing whether the new model is feasible for implementation, finding and incorporating solutions to the barriers.

The benefits of this co-designed model will be holistically evaluated considering the priorities of national health care strategy and PPI perspectives. This study will result in a set of guidelines and recommendations for the implementation of this model of care nationally.

## Methods/design

**Figure 1.**
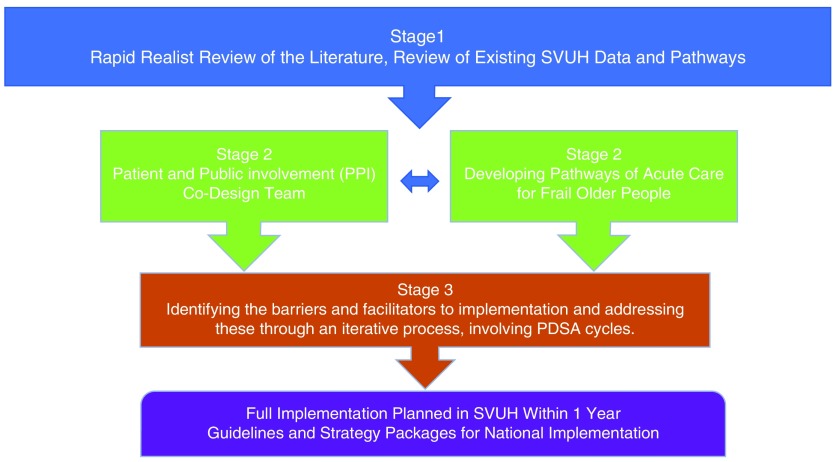
SAFE research plan.

The SAFE project has been funded by the Irish Health Board applied partnership award (APA) and the grant specifications have influenced the design of the study ( Figure 1).

The HRB APA, the first grant of its kind, which stresses the research findings must be applied by the knowledge user organizations ^29^. The award supports projects where academic researchers and knowledge users coming together in a collaboration to focus on themes/questions which are determined by the documented needs of the Irish health and social care system.

## Ethical considerations

Ethical approval to conduct the study was given approval provided by St. Vincent’s Healthcare Ethics and Research Committee on the 15 ^th^ of February 2017 (Ref SAFE: 23/2/17) and from University College Dublin Human Research Ethics Committees on the 23 ^rd^ of January 2017 (LS-17-05-ODonnell). Informed written consent will be obtained for all participants in the co-design workshops and for interviews.

## Study setting SAFE partnership approach

**Figure 2.**
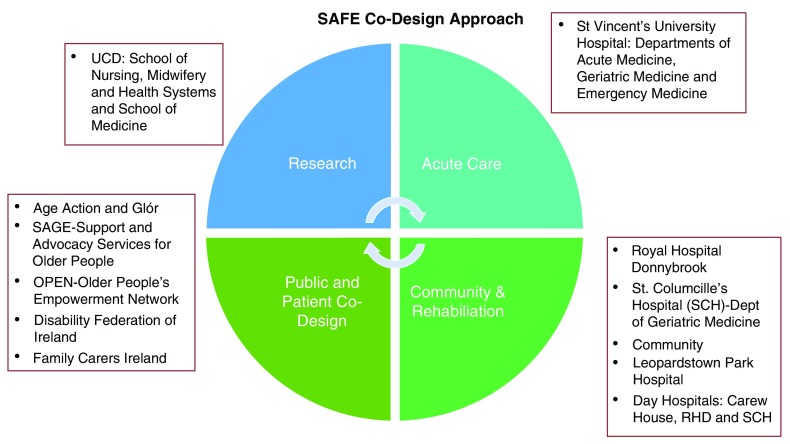
SAFE co-design approach.

This study will be conducted primarily in SVUH which is a large acute teaching hospital located south of the city of Dublin. SVUH is part of the Ireland East Hospital Group (IEHG) which was established in January 2015. IEHG is the largest of the hospital groups and serves a population catchment area of over 1.1 million. Recognising this, our approach involves not only the acute setting of SVUH but includes our partners within the community and rehabilitation sector, members of the public and researchers ( Figure 2).

These identified partners will ensure that the developed pathway includes all relevant stakeholders to ensure sustainability of all stages of the research. 

## Rapid realist review of the literature

A rapid realist review (RRR) is proposed for the literature review which is a relatively new approach to synthesis knowledge ^30^. For healthcare staff working within the health system a RRR is an approach that aids the unpacking of the contexts and interrelated mechanisms underlying implementation activities ^31– 33^ *.* More broadly the benefits of engaging diverse stakeholders in the co-production of the literature review process is often stressed as being advantageous as it provides increased clarity and awareness of the transferability of the review findings ^31, 33^. A full realist review undertakes a long process of exploring the literature and includes a period of ‘testing’ programme theories. In contrast, an RRR has emerged to assist a speedier transition from research to practice. RRR’s are particularly useful during the initial phase of a multiphase project where research findings need to be rapidly adapted to take account of emerging evidence and where there is limited time and resources ^34, 35^. The basic question of an RRR is ‘what is it about this intervention that works in this context and why?’ ^33^. An RRR works on understanding of the factors enabling and constraining the implementation of a particular intervention ^35^.

Key to the success of a RRR is the engagement of expert and reference panels. Reference panels provide local knowledge ensuring that the review is sensitive to the experience of those ‘on the ground’ ^35^. The expert panel is composed of content experts who are tasked with ensuring that the review undertaken reflects current thinking. They are also expected to make available grey and or operational documentation not yet published and share relevant academic literature to tailor the search strategy. The inception meeting of the expert panel will agree the research question currently proposed as ‘what are the factors that enable the successful implementation of a frail older person’s pathway in the acute setting’.

To ensure that the review is sensitive to those locally we propose two reference panels which will be reviewed by the expert panel. Our reference panel proposal will be established via two processes. The first will be online using social media. We propose using the #IrishMed which is a live twitter-driven event which takes place every Wednesday for an hour at 10 pm Irish time on all things relating to medicine. On average between 180 to 200 participants take part, sending between 1500–2000 tweets. Weekly hosts facilitate the discussion by providing four to five questions to direct the conversation in areas such as eHealth, Integrated Care and pain management ^36^. This is an innovative approach that will enable the capturing of the experiences from those working and accessing health care system both in Ireland and internationally. The discussion questions during the curated chat will focus upon:

- What mechanism/resources enabled the development and implementation of a frail older person pathways intervention to be successfully implemented within the acute setting?
- What were the underlying contextual conditions that activated these mechanisms?

Our second reference panel process will involve hosting a workshop with our public and patient co-design panel. The outcome of the reference panel process will be summarised into the initial programme theory developed.

Data will be extracted over 8–10 weeks and weekly data sessions will be held to critically appraise, analyse and synthesise the data. Two members of the team (MTC and ÉNS), in consultation with a university faculty librarian will undertake a search of the literature using PubMed, Cinahl and Web of Science databases. These databases are suggested as they offer extensive and complementary indexing of relevant literature. Other databases will be reviewed and agreed by the expert panel. This search will also be supplemented with key articles and other documents relevant from the grey literature as identified by the group. The search for evidence in a RRR is iterative, and will be progressively extended and refocused based on the identified sources as the review evolves.

Following agreement of the expert/reference group the RRR will identify mechanisms (individual or collective) that were observed across different interventions leading to successful implementation. Contextual factors will also be identified that enabled or inhibited the activation of specific mechanisms. The results of the synthesis will be written up adopting the ‘Realist and Meta-Review Evidence Synthesis: Evolving Standards’ (RAMESES) standard for reporting realist reviews ^37^. The results of this review will provide the foundation for the co-design stage of the program.

## Overview of current pathways in St Vincent’s University Hospital

A review of SVUH emergency department data for 2014 and 2015 will be undertaken to capture baseline data of those aged over 65 years attending. This de-identified data set will be analysed using Stata 14 Statistical Software Package by the research team to capture the following baseline date:

1. Age profile of ED visits focusing on persons over 65s

2. Types of entry by this cohort –e.g. ambulance, walking etc.

3. Where patients came from e.g. GP referral, home, nursing home

4. Whether these patients were admitted and to what ward

5. Whether there were repeat presentations / re-admissions

6. Length of time in the ED and where did they go to.

7. Clinical outcomes

Semi-structured interviews will be undertaken with key staff in SVUH including the ED triage nurse, relevant health care & social care professions, doctors, consultants, bed management and senior management (such as assistant directorate of nursing) and with relevant partners in the community and rehabilitation setting. This will inform the documentation of the pathway as it currently stands, the problems and bottlenecks in the systems and opinions and insights into how these could be improved. This information will be recorded, transcribed, de-identified and coded. The codes will be translated into trends and themes. Patterns will be recognised using qualitative methods research including NVivo V11 software. This analysis will increase our understanding of the advantages and problems with the current model of care.

## Co-design of integrated care pathways public and patient involvement

Public and patient involvement (PPI) has been a key consideration in healthcare for several decades now. Whilst the literature is clear in stressing the merits of engaging PPI partners ^38^ very little evidence is available as to how these are undertaken and what the best approaches are ^39, 40^. A significant focus of the SAFE project is that the intervention will be co-designed by the team in collaboration with members of the public. Our approach to co-design involves developing democratic partnerships between researchers and PPI stakeholders with a view to involving them in the design of research, promoting their understanding and capacity, and encouraging uptake of findings ^41^. Six PPI group members will be recruited through networks already established by the research team and will ensure that the interventions will be grounded in the needs and perspectives of the target users and complemented by the expertise of the research team. The user group will be modelled on the Older People’s Empowerment Network (OPEN), which was established in 2013 by co-author DO’D and colleagues when developing the Keep Control intervention to enable older people to protect themselves from older person’s financial abuse ^42^.

PPI members will participate in a total of six co-design workshops of approximately 2 hours’ duration each. These workshops will be co-chaired by DO’D and research team participants will include co-authors MTC, DO’D, ÉNS, EA, AP and a SVUH clinical nurse specialist in geriatrics (to be appointed). Input from healthcare professionals will assist the co-design participants in understanding project objectives and how the system of care for older people currently functions from the perspective of service delivery. The first four co-design workshops will run in parallel to a SVUH pathway development component of the project and will consist of:

(1) Introductions and review of project aims and objectives. Presentation to the group of the initial scoping of the rapid realist review of the literature and presentation of current SVUH pathways.
(2) In-depth discussion and workshopping of data focussed on PPI experiences and understandings of frailty in later life as well as care of older people
(3) Consolidation of PPI recommendations and adaptations to the proposed model care pathway
(4) Review of patient-centred outcomes for pathway evaluation.

Additional workshops may be added as required and requested by the co-design participants. It is anticipated that this phase of the co-design activities will result in a model for care of frail older people which is patient-centred and grounded in the needs and perspectives of target users. The final two co-design workshops will run alongside the testing of the pathway and will assist with the PDSA process testing of the new pathway ensuring patient-centred outcomes are employed in the identification of barriers and enablers to successful pathway implementation. These workshops will be structured as follows:

(5) Assessment of patient-centred outcomes of co-designed redevelopment of pathways within SVUH for the frail older patient further redefined
(6) Reviewing and contributing to the recommendations for successful implementation of care pathway.

Summary notes from each workshop will be typed up by the research team and fed back to the participants for review and sign off. **

## Identifying the barriers and facilitators to implementation and addressing these through an iterative process

Following the identification of outcomes and processes to include in the pathway successive rounds of Plan-Do-Study-Act (PDSA) cycles will be undertaken. The benefit of a PDSA method lies in the learning that occurs and enables the opportunity to undertake adjustments accordingly. This is a suitable process to use for a co-design approach and factors the complexity of a health system. The flexibility and adaptability of PDSA cycles are important features that support the adaption of interventions to work in local settings ^43^. It is anticipated that several cycles of PDSA will occur to test the co-design frail older person’s pathway. During each cycle which will be undertaken by overseen by a clinical nurse specialist in geriatrics short interviews will be undertaken with patients and staff will be used to assess the performance of the new system in terms of outcomes.

The different elements in the pathway will be introduced tested on a phased basis, individually initially, then combined. The testing will be for very short timeframes initially, and successively lengthening as the process evolves. The systems for care of frail older people will be continually evolved through a process of assessing the outcomes and addressing deficiencies. Following consultation with our PPI co-design group goals which are not being met will lead to changes in process to rectify the problem. Through this iterative process, we aim to ensure that the solution meets not only goals which are important in terms of national strategy but the aspects which are important to patients, providing a more patient centred solution.

## Discussion

It is anticipated that the development of a frailty pathway, based on international experience and tailored to the context of the Irish healthcare setting through extensive co-design work, will deliver results across the system. The establishment of a template for care of the frail older people in Ireland will be an important development and is clearly aligned with the National Clinical programmes, healthcare policy and national/international guidelines. Careful attention has been paid in the study design to tailor the pathways to allow the pathways to be implemented quickly and effectively in the local and afterwards the national setting. Most importantly, the pathways will provide patient-centred and integrated care by embedding a co-design approach.

It is the expectation of the knowledge user organisation SVUH that this pathway would then be implemented within a year of this study. Importantly the SAFE study will have laid the groundwork for the pathway to be assessed in terms of achieving outcomes and appropriate implementation. The eventual outcome will be nationally relevant guidance on implementation of patient centred pathways for frail older people, in the Irish context, which meet standards mandated in national policies. Key to the success of the study will be the engagement of hospital staff and clinical leaders as integral stakeholders and participants in this programme of work.

## Dissemination of results

The opportunity to disseminate the findings from this study across the Ireland East hospital group is considerable, and the findings from this study will be disseminated to the research community through publications in international peer-reviewed journals and presentations at international conferences on geriatric care and on quality and safety. In consultation with our PPI co-design group patient advocacy groups will also disseminate the findings to patients and the public through their websites, patient newsletters and information evenings.

**Study status:** As of February 2018 stage one of the SAFE study has been completed. Stage two, the co-design of the integrated care pathway is near completion but was delayed due to the significant pressures on SVUH over the winter period. Stage three is due to commence in March 2018.

## Ethics approval and consent to participate

Ethical approval to conduct the study was given approval provided by St. Vincent’s Healthcare Ethics and Research Committee on the 15 ^th^ of February 2017 (Ref SAFE: 23/2/17) and from University College Dublin Human Research Ethics Committees on the 23 ^rd^ of January 2017 (LS-17-05-ODonnell). Informed consent will be obtained for all participants in the co-design workshops and for interviews.

## Data availability

*All data underlying the results are available as part of the article and no additional source data are required*
