# Supplementary material for: The systematic approach to improving care for Frail Older Patients (SAFE) study: A protocol for co-designing a frail older person’s pathway
Source: HRB Open Res. 2018 May 8;1:9. Originally published 2018 Mar 5. [Version 2] doi: 10.12688/hrbopenres.12804.2 (PMC6973535; doi:10.12688/hrbopenres.12804.2)
Supplement: Peer review history

# Peer review: Reviewer response for version 2

**DOI:** 10.21956/hrbopenres.13894.r26214

**Published:** 2018-05-08

**Authors:** Adam Gordon

**Affiliations:** 1 Division of Medical Sciences and Graduate Entry Medicine, University of Nottingham, Nottingham, UK; 2 City, University of London, London, UK

I am comfortable that the authors have addressed my concerns in their amendments. The RRR section of the article is now much more explicit about the work which will be conducted.  It is also clear now how this will feed into the subsequent work as the authors state that the results of RRR will be presented at the co-design workshops.

 I wonder if the RRR might also give the authors an analytical framework through which to consider the operation of their co-design workshops.  But it is reasonable that they have not chosen to state this explicitly as to do so might make them something of a hostage to fortune if the findings of their RRR don't provide a practical or useful analytical framework.

 I now feel that this protocol paper can be published without further amendment.

I confirm that I have read this submission and believe that I have an appropriate level of expertise to confirm that it is of an acceptable scientific standard.

# Peer review: Reviewer response for version 1

**DOI:** 10.21956/hrbopenres.13864.r26129

**Published:** 2018-04-30

**Authors:** Adam Gordon

**Affiliations:** 1 Division of Medical Sciences and Graduate Entry Medicine, University of Nottingham, Nottingham, UK; 2 City, University of London, London, UK

This is a mostly well-written protocol which attempts to summarise a complex, multi-phase study which brings together components of realist synthesis, co-design methodology and quality improvement methodology (in the form of Plan-Do-Study-Act cycles).  The project is ambitious and commendably straddles the boundary of research and QI.  This is true implementation research.

 I have three main criticisms, as follows:

1. The flow of knowledge through the programme, from realist synthesis to co-design to QI sections is not clear.
2. The realist synthesis section is, I feel, under-written.
3. The ways in which knowledge will generated to make it generalisable outside of the hospital in which it has been developed are not clear.

I will consider these points in turn below:

1. Flow of knowledge: to make this programmatic, rather than just a constellation of separate projects, the flow of knowledge between each of the components needs to be clear.  I am particularly concerned that the realist review, once done, doesn't seem to inform subsequent pieces of work.  The ultimate goal of the realist synthesis will be to develop a programme theory.  Once this has been generated it could, for example, provide a theoretical framework either for structuring, or interpreting, the outputs of the co-design work. At the moment, though, this is not clear.  I am sure that the authors have in mind some utility for the outputs from their realist synthesis with regard to later stages of the programme but at present it is not evident in their protocol.
2. The realist synthesis section is, I think, at present underwritten.  A realist review should generate more than a list of barriers and facilitators.  It should generate a programme theory of what works under what circumstances to achieve outcomes of interest.  I think the authors need to express more clearly what the outcomes of interest are - this will enable them to conduct their realist review more concisely and for the reader of the protocol to better understand their frame of interest.  I have only done traditional realist reviews and not RRRs but my understanding is that a challenge with RRRs is keeping the focus sufficiently narrow to ensure that the literature can be interrogated with sufficient depth within the available time and resource.  I would have more confidence of the authors' ability to do this if the outcomes of interest were specified.  A key component of realist synthesis is looking for recurrent patterns within the evidence, demiregularities in Context-Mechanism-Outcome (CMO) configurations, that form the basis of a programme theory.  This does not come through in the protocol and I would like the authors to provide more detail on how they will come to a data-driven programme theory.  Finally, the use of social media is innovative but I'm left wondering, due to the lack of detail, whether it might be a slightly naiive inclusion.  How will social media inform the emerging CMOs, or the programme theory based upon them? How will responses relevant to emerging theoretical framework be solicited on social media and how will the data be analysed, interpreted and integrated into the theoretical framework with appropriate weighting?  I really need more detail here to be confident that this is something other than a wheeze which looks good on paper.
3.  At the moment, the only part of the programme which looks convincingly like it will generate knowledge generalisable outside of the hospital where the research is being conducted is the realist review.  Co-design and PDSA cycles are notoriously context -dependent and if the researchers are not careful they will simply develop a wonderful service for their hospital without being able to generalise these findings to other settings.  I think one way to tackle this would be tackling points 1. and 2. above.  If the realist synthesis generated a programme theory which informed the co-design and the PDSA, and was subsequently iterated in response to the findings from these components of the research programme, then this would result in a finalised programme theory which could guide implementation in settings outside of St Vincents. The authors might then give emphasis to this issue of generalisability in their Discussion.  If they conveyed such an approach more clearly in their protocol I would be more convinced that they were using co-design and PDSA as research tools, rather than just doing a bit of research (realist synthesis), followed by a piece of QI (co-design and PDSA).

This is good work and I mean these comments to be taken as a constructive opportunity for the authors to better sell the benefits of their research programme.  I hope they take my comments in this way.

I confirm that I have read this submission and believe that I have an appropriate level of expertise to confirm that it is of an acceptable scientific standard, however I have significant reservations, as outlined above.

# Response

**Published:** 2018-05-04

**Authors:** Éidín Ní Shé

**Affiliations:** University College Dublin, Ireland

Dear Adam, On behalf of the SAFE team we wish to thank you for the review and for the comments you have provided. We wish to reply to the following:

 Point 1 & 2 Combined: Thank you for the feedback on the rapid realist review- A full realist review undertakes a long process of exploring the literature and  includes a period of ‘testing’ programme theories. In contrast an RRR, has emerged to assist a speedier transition from research to practice. We have reviewed the suggestions you have made and expanded the section as you suggested providing clarity on the reference panel process and outlining how the RRR will provide the basis of the pathway development.

 Point 3: The SAFE study is funded by the HRB Applied Partnership Awards (APA)-The focus of the call is to specifically: support high quality applied research projects where academic researchers and knowledge users come together in a collaboration to focus on themes/questions which are determined by the documented needs of the Irish health and social care system. Documented needs are defined as ’to the research priorities or needs of the lead knowledge user applicant'. Co-design and PDSA cycles are thus appropriate methods ensuring that the pathway that is developed captures and responses to the local context.  We are working to ensure that the pathway developed will be disseminated across the Ireland East Hospital Group (comprising 11 hospitals) and via our Public and Patient partner organisations.

# Peer review: Reviewer response for version 1

**DOI:** 10.21956/hrbopenres.13864.r26125

**Published:** 2018-03-19

**Authors:** Roman Romero-Ortuno

**Affiliations:** 1 Department of Medicine for the Elderly (DME), Addenbrooke's Hospital, Cambridge, UK; 2 Clinical Gerontology Unity, Department of Public Health and Primary Care, University of Cambridge, Cambridge, UK

This protocol presents a comprehensive overview of an ambitious -but realistic- quality improvement protocol that specifically aims at improving the integrated frailty pathway in a well known major university hospital in South Dublin (SVUH).

 The protocol is very well written and reflects the emphasis on an integrated co-design approach to the quality improvement protocol. This is also reflected in the composition of the research team, with a very good mix of front-line acute clinicians, intermediate and community clinicians, clinical academics, policy stakeholders, and public and patient involvement. The mixed quantitative and qualitative approach is very beneficial to the research questions and design.

 This study will be conducted primarily in SVUH and the authors anticipate that lessons learned will be able to influence national policy. The group is very well positioned to achieve that aim.

 This piece of research is highly novel and valuable in the Irish setting and the policy translation of the findings could be very important in order to provide an evidence-based response to the challenges that acute hospitals are facing in developed countries given demographic trends. The authors may consider, in future iterations of the research, whether lessons learned locally can also be extrapolated to other Irish centres before they can be recommended nationally. It may be important to differentiate lessons that are dependent on local context and require mostly local change from lessons that are system-dependent and  require a national policy response. Collaboration with other Irish centres in future iterations may be a very welcome way forward in order to help translate common theme learnings into national policy.

I confirm that I have read this submission and believe that I have an appropriate level of expertise to confirm that it is of an acceptable scientific standard.

# Response

**Published:** 2018-05-04

**Authors:** Éidín Ní Shé

**Affiliations:** University College Dublin, Ireland

Dear Roman, Thank you for the feedback. We will ensure that our focus on sharing the results of the SAFE study will be a central part of the work. We have already commenced this by hosting a research showcase of the work undertaken thus far but advance this further.
